# Upregulation of outer membrane porin gene *ompC* contributed to enhancement of azithromycin susceptibility in multidrug-resistant *Escherichia coli*

**DOI:** 10.1128/spectrum.03918-23

**Published:** 2024-03-05

**Authors:** Xing-Wei Luo, Peng-Liang Li, Ya-Jun Zhai, Yu-Shan Pan, Gong-Zheng Hu, Dan-Dan He

**Affiliations:** 1 College of Veterinary Medicine, Henan Agricultural University, Zhengzhou, China; Yangzhou University, Yangzhou, China

**Keywords:** azithromycin, membrane, *ompC*, *mph*(A), conformation

## Abstract

The outer membrane (OM) in gram-negative bacteria contains proteins that regulate the passive or active uptake of small molecules for growth and cell function, as well as mediate the emergence of antibiotic resistance. This study aims to explore the potential mechanisms for restoring bacteria to azithromycin susceptibility based on transcriptome analysis of bacterial membrane-related genes. Transcriptome sequencing was performed by treating multidrug-resistant *Escherichia coli* T28R with azithromycin or in combination with colistin and confirmed by reverse transcription-quantitative PCR (RT-qPCR). Azithromycin enzyme-linked immunosorbent assay (ELISA) test, *ompC* gene overexpression, and molecular docking were utilized to conduct the confirmatory research of the potential mechanisms. We found that colistin combined with azithromycin led to 48 differentially expressed genes, compared to azithromycin alone, such as downregulation of *tolA*, *eptB*, *lpxP*, and *opgE* and upregulation of *ompC* gene. Interestingly, the addition of colistin to azithromycin differentially downregulated the *mph*(A) gene mediating azithromycin resistance, facilitating the intracellular accumulation of azithromycin. Also, overexpression of the *ompC* elevated azithromycin susceptibility, and colistin contributed to further suppression of the Mph(A) activity in the presence of azithromycin. These findings suggested that colistin firstly enhanced the permeability of bacterial OM, causing intracellular drug accumulation, and then had a repressive effect on the Mph(A) activity along with azithromycin. Our study provides a novel perspective that the improvement of azithromycin susceptibility is related not only to the downregulation of the *mph*(A) gene and conformational remodeling of the Mph(A) protein but also the upregulation of the membrane porin gene *ompC*.

**IMPORTANCE:**

Usually, active efflux via efflux pumps is an important mechanism of antimicrobial resistance, such as the AcrAB-TolC complex and MdtEF. Also, bacterial porins exhibited a substantial fraction of the total number of outer membrane proteins in Enterobacteriaceae, which are involved in mediating the development of the resistance. We found that the upregulation or overexpression of the *ompC* gene contributed to the enhancement of resistant bacteria to azithromycin susceptibility, probably due to the augment of drug uptakes caused and the opportunity of Mph(A) function suppressed by azithromycin with colistin. Under the combination of colistin and azithromycin treatment, OmpC exhibited an increased selectivity for cationic molecules and played a key role in the restoral of the antibiotic susceptibility. Investigations on the regulation of porin expression that mediated drug resistance would be important in clinical isolates treated with antibiotics.

## INTRODUCTION

Antimicrobial resistance in bacteria is a widespread and ongoing threat, which weakens the effectiveness of drugs that are even the most efficient bactericidal agents. Investigating regulation mechanisms in gene levels of antimicrobial susceptibility and exploring new targets of drugs are the fundamental steps of the resistance. It was reported that the genes contributed to conferring resistance by controlling the intracellular levels of drugs, which encoded the outer membrane (OM) porin and the multidrug efflux pump (1). In Enterobacteriaceae, trimeric porins of the OM control the intracellular uptake of small molecules, such as nutrients and antibacterial agents. However, the active efflux via efflux pumps in the inner membrane and the relatively slow passive uptake mediated by the porins across the OM cause a permeability barrier, efficiently reducing the intracellular concentrations of drugs and further facilitating the development of resistance (2).

Previous studies suggested that azithromycin in combination with colistin represented bactericidal activity against colistin-resistant strains (3). Although azithromycin that is combined with colistin showed the synergistic activity *in vitro* and *in vivo*, enhancement mechanisms of azithromycin susceptibility remain poorly understood. In this study, a representative strain was employed to explore the potential mechanisms of restoral azithromycin susceptibility, which was resistant to both azithromycin and colistin.

## RESULTS

### Regulation of membrane-related genes in response to the combination treatment

**Fig 1:**
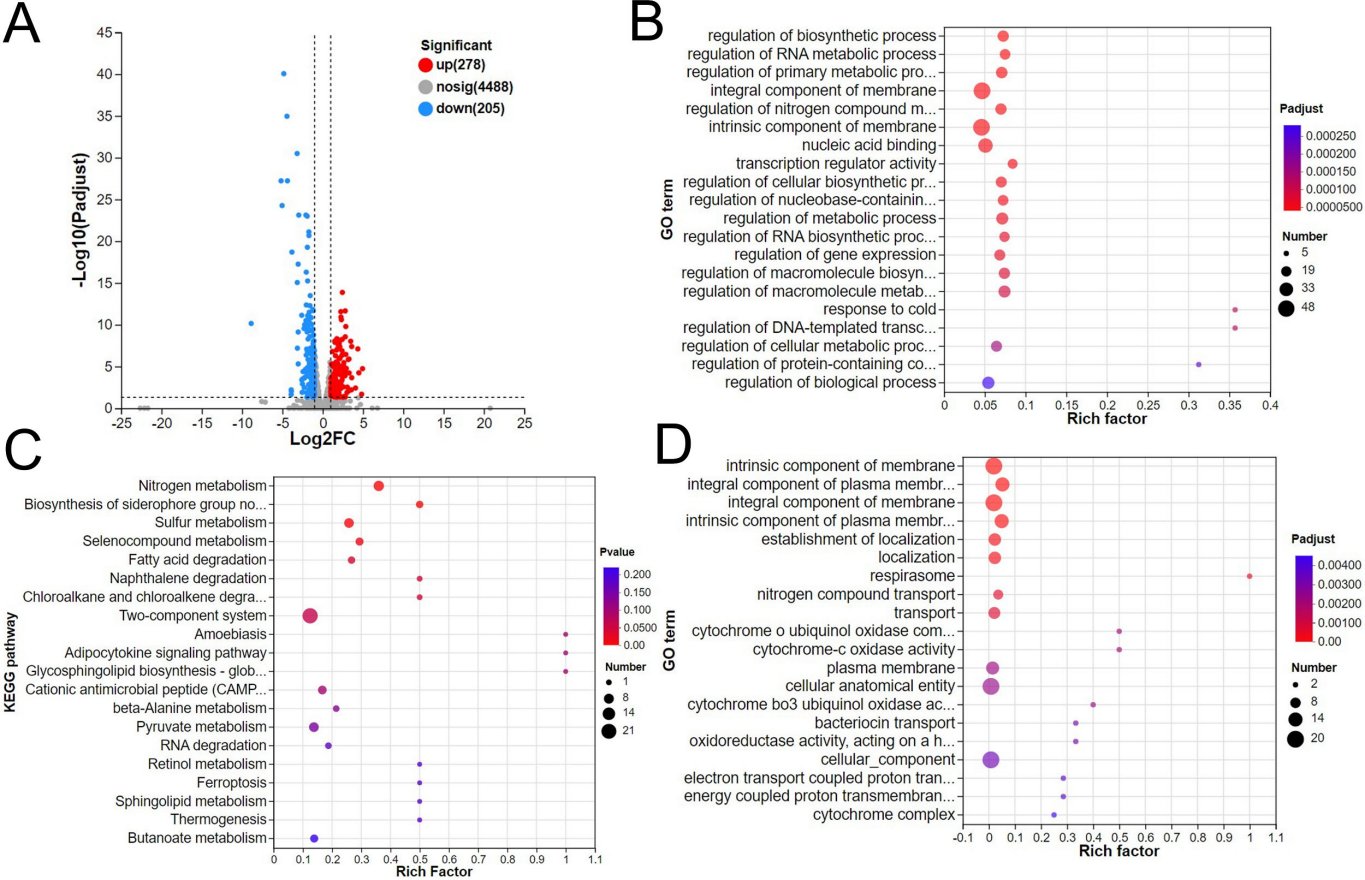
Transcriptomic profile of *E. coli* T28R treated with azithromycin and the combination of azithromycin and colistin. (**A**) Volcano plot. The combination led to 278 upregulated and 205 downregulated differential expression genes. (**B**), (**C**), and (**D**) GO and KEGG enrichment analyses of DEGs.

A comparative transcriptomic analysis reveals differentially expressed genes (DEGs) that are regulated by the addition of colistin to azithromycin. To explore the molecular mechanisms of azithromycin resistance reversed by colistin, the transcriptomic profile of *Escherichia coli* T28R in response to azithromycin plus colistin was mapped to that treated with azithromycin alone. As a result, a total of 278 upregulated and 205 downregulated DEGs were identified, which were involved in biological processes such as regulation of biosynthetic process, cellular components such as integral component of membrane, and molecular functions such as transcription regulator activity according to a gene ontology (GO) enrichment analysis (Fig. 1A and B). Some of these genes were related to nitrogen metabolism, fatty acid degradation, and two-component system by a Kyoto Encyclopedia of Genes and Genomes (KEGG) cluster analysis (Fig. 1C). Interestingly, there were 48 DEGs related to the integral component of the membrane being further analyzed by GO, suggesting that these DEGs mediated the integrity of membrane, transport, and electron transport-coupled proton transport (Fig. 1D).

**Fig 2:**
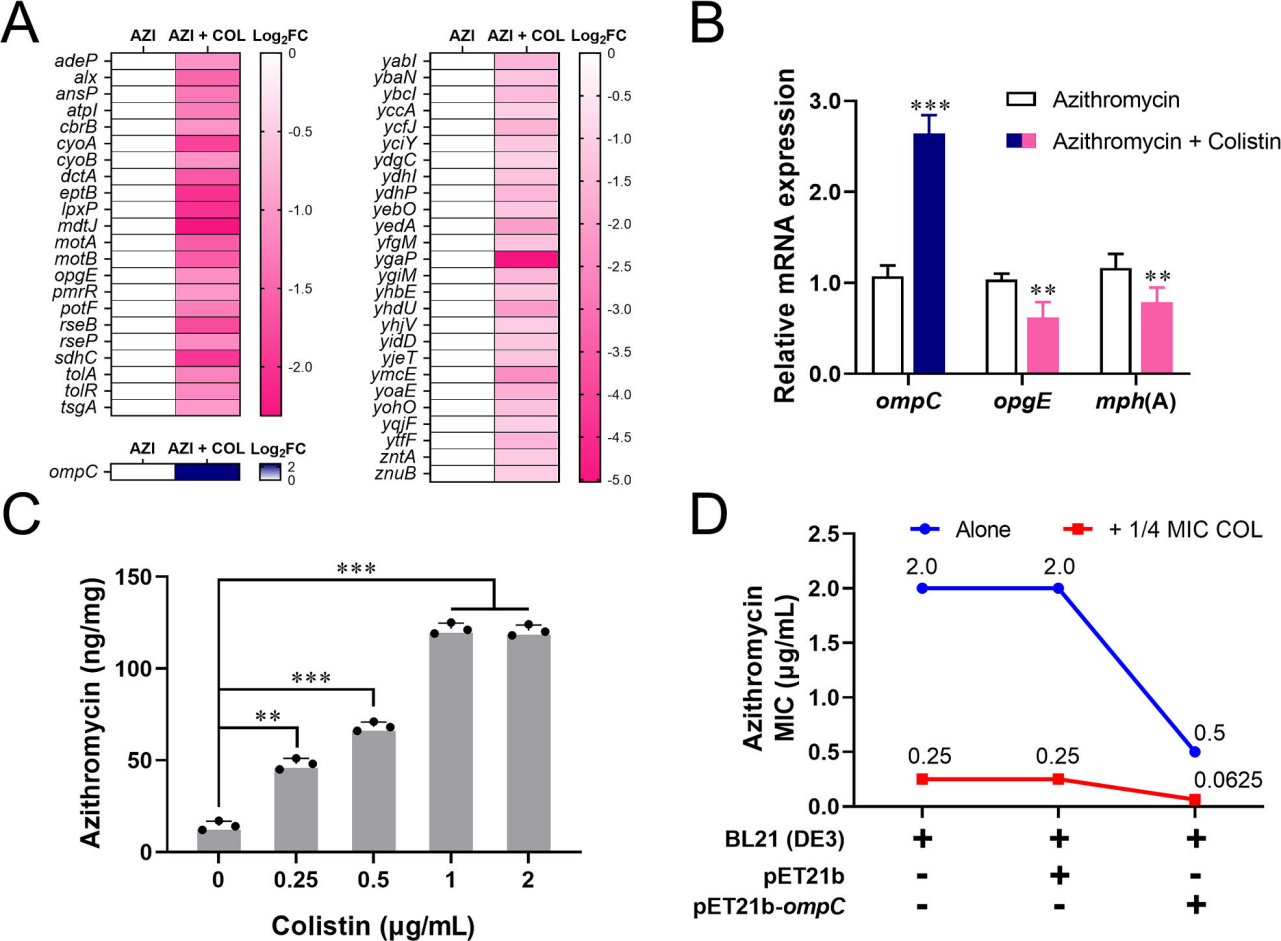
The mechanisms of restoral of azithromycin susceptibility. (**A**) Colistin combined with azithromycin led to membrane-related differential expression genes including downregulation of the *tolA*, *eptB*, and *lpxP* and upregulation of the *ompC*, compared to azithromycin alone. (**B**) RT-qPCR analysis of the *ompC*, *opgE*, and *mph*(A) genes treated with colistin combined with azithromycin, compared with azithromycin alone. (**C**) Colistin contributed to the accumulation of azithromycin in *E. coli* T28R. (**D**) Overexpression of the *ompC* gene in *E. coli* BL21 (DE3) resulted in a more decreased azithromycin MIC, especially in the presence of colistin. AZI, azithromycin; AZI + COL, azithromycin combined with colistin. Data are presented as mean ± SD and unpaired *t*-test was used to calculate *P* values (*P < 0.05, **P < 0.01, and ***P < 0.001).

Specifically, the *tolA* gene-encoding cell envelope integrity protein and *tolR*-encoding colicin uptake protein were significantly downregulated, suggesting a biological disturbance of membrane component, which was barely reported (Fig. 2A). The transcriptomic levels of the genes involved in lipopolysaccharide (LPS) modification were observably downregulated such as *eptB* and *lpxP* genes, which encoded respectively kdo (2)-lipid A phosphoethanolamine 7″-transferase and kdo (2)-lipid IV(A) palmitoleoyltransferase, indicating the modification of LPS lipid A was interrupted. These results suggested that colistin can have a negative effect on the stability of the membrane in *E. coli* T28R after binding to the LPS of the OM, facilitating the enhancement of membrane permeability (4).

Under azithromycin plus colistin pressure, the genes related to cell motility including *motA*-encoding flagellar motor stator protein and *motB*-encoding flagellar motor protein were also downregulated (Fig. 2A), suggesting a slow mobile trend of the cells. This is corresponding to significant downregulation of the genes *cyoA* and *cyoB* that are involved in the electron transport chain, and *sdhC* that is related to oxidative phosphorylation, indicating a disturbed energy production. Additionally, the genes associated with C4-dicarboxylate transporter DctC (*dctA*) and drug/metabolite exporter (*yedA*) were remarkably downregulated. Notably, some OM-related genes, including the *ompC* gene encoding a porin protein (Fig. 2A), were significantly upregulated, which facilitated exhibiting selectivity for cationic molecules (2). The abovementioned DEGs were proved by reverse transcription-quantitative PCR (RT-qPCR) assays, showing a consistent expression trend in comparison with transcriptome analysis (Fig. 2B; Fig. S1).

### The addition of colistin led to downregulation of *mph*(A) gene

Importantly, the gene *opgE*, encoding a phosphoethanolamine transferase, showed a significant downregulation in response to azithromycin plus colistin (Fig. 2A and B). Some non-synonymous mutations of *opgE* related to lipid A modification conferred resistance to colistin (5). So, the *opgE* downregulation was an interesting response to synergistic activity of azithromycin combined with colistin against the isolate T28R. Additionally, the *mph*(A), encoding macrolide 2′-phosphotransferase and mediating azithromycin resistance (6), was downregulated under the treatment of the combination, consistent with RT-qPCR results (Fig. 2B), which inferred that this is a key cause of restoral of azithromycin susceptibility.

### Colistin caused the intracellular accumulation of azithromycin

It is necessary for sufficient accumulation of antibiotics in cells to exhibit antibacterial activity (7, 8). By conducting an enzyme-linked immunosorbent assay (ELISA) assay, we found that azithromycin content was accumulated in an approximately dependent manner of colistin dose in *E. coli* T28R (Fig. 2C). The intracellular accumulation of azithromycin gradually rose when colistin was added ranged from 0 to 1 µg/mL but was not further elevated in the presence of 2 µg/mL, which could be related to bacterial viability. To some extent, the result suggested that colistin helps retention of azithromycin in cells.

### Overexpression of *ompC* gene elevated azithromycin susceptibility

Furthermore, the *ompC* gene was cloned into plasmid pET21b and transformed into *E. coli* BL21 (DE3) to explore its role. As a result, overexpression of *ompC* gene in *E. coli* BL21 (DE3) led to a decreased MIC of azithromycin (Fig. 2D). Compared with BL21 (DE3) and BL21 (DE3) + pET21b, MIC of azithromycin against BL21 (DE3) + pET21b-*ompC* was 0.5 µg/mL and decreased by four times, which indicates that *ompC* gene mediated the enhancement of azithromycin susceptibility. Especially, azithromycin MIC against BL21 (DE3) + pET21b-*ompC* was changed into 0.0625 µg/mL in the presence of 1/4 MIC colistin, which was decreased by 32 times in comparison with the alone MIC against BL21 (DE3) or BL21 (DE3) + pET21b. These data suggested that the synergy of *ompC* upregulation with colistin mediated positively the intracellular uptake of azithromycin and contributed to enhanced susceptibility of bacteria to azithromycin.

### Colistin facilitated remodeling of the mph(A) protein

**Fig 3:**
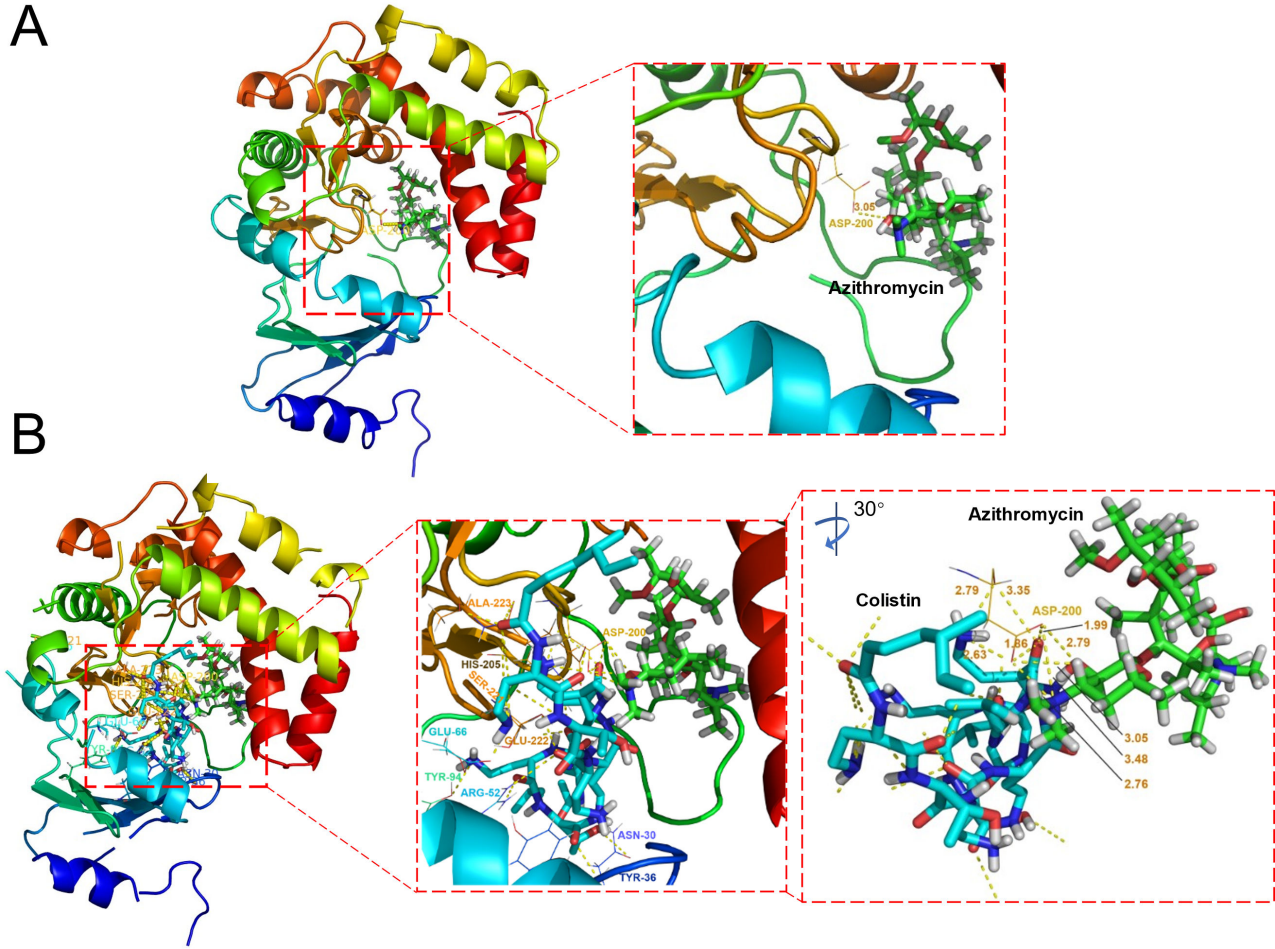
Three-dimensional structural view of molecular docking. (**A**) Conformational analysis of the Mph(A) protein containing azithromycin ligand (5IGI). (**B**) The binding model of colistin and the Mph(A) protein in the presence of azithromycin ligand (5IGI). Red dashed rectangles magnified showed the specific interacting modes between drugs and amino acid residues in the docking pocket. Yellow dashed represents hydrogen bonds. ASP, aspartate.

To analyze the binding mode between colistin alone and colistin combined with azithromycin and Mph(A) protein, we performed molecular docking by using the drugs to fit in the docking pocket of Mph(A). By visualizing analyses, we found that the Mph(A) protein (5IGI) showed a simple binding mode with azithromycin molecular, only forming a hydrogen bond with 3.05 Å between aspartate residue ASP-200 of the Mph(A) and azithromycin (Fig. 3A). However, the addition of colistin led to a more complex mode with many residues and hydrogen bonds, next to azithromycin (Fig. 3B). Interestingly, the ASP-200 formed five hydrogen bonds with colistin ranging from 1.86 to 3.35 Å and formed four hydrogen bonds with azithromycin ranging from 2.76 to 3.48 Å. The phenomenon indicated that colistin competed with azithromycin for the activity pocket of Mph(A). However, colistin alone interacting with Mph(A) (5IGH) showed fewer hydrogen bonds in the docking pocket with other residues (Fig. S2). These results suggested that the addition of colistin more conveniently changed the Mph(A) protein conformation, like forming a more stable and tight interacting mode with Mph(A), conceivably exhibiting a repressive effect on its phosphotransferase activity.

## DISCUSSION

The emergence and development of antimicrobial resistance in pathogenic bacteria is one of the major issues that currently pose a severe threat to public health. For azithromycin resistance, the potential molecular mechanisms have been described, such as overexpression of efflux pump or peptidyl tRNA hydrolase, ribosomal protein or *23S rRNA* mutation, methylation mediated by methylases encoded by *erm* genes, esterase-mediated inactivation including EreA and EreB, and phosphotransferases encoded by *mph*(A) and *mph*(B) (6, 9). Recently, some studies suggested that azithromycin resistance is mainly mediated by the genes frequently carried on mobile genetic elements such as *mph*(A), *erm*(42), *erm*(B), and *ramAp* gene (10, 11). Probably, the related determinants of azithromycin susceptibility are still needed to be further investigated.

After exposure to antibiotic, efflux pumps are commonly overexpressed and can export chemically various compounds, causing a lower cellular antibiotic concentration and then drug resistance (12). However, in this study, we found that the combination of colistin and azithromycin restored the susceptibility of multidrug-resistant *E. coli* to the antibiotics, being characterized by *ompC* gene upregulation and *mph*(A) downregulation, compared to azithromycin-alone treatment. With further investigation, the overexpression of *ompC* decreased azithromycin MIC against *E. coli*, indicating a significant effect on azithromycin susceptibility, especially for the more decreased MIC in the presence of 1/4 MIC colistin, which differed from a previous report that azithromycin mediated the expression of efflux pumps (13). For this reason, the decreased azithromycin MIC caused by *ompC* regulation might reflect the fact that the *ompC* partly facilitated the intracellular uptake of azithromycin to some extent. Correspondingly, previous studies suggested that the loss of OmpC in *E. coli* promoted antimicrobial resistance such as carbapenems and fourth-generation cephalosporins and a higher mutation rate of the *ompC* gene was detected in multidrug-resistant strains (14). Additionally, some membrane-related genes such as *tolA*, *eptB*, and *lpxP* showed differential downregulation, suggesting a damaging trend of the membrane function. Indeed, colistin contributed to the intracellular accumulation of azithromycin by the ELISA assay, which inferred that colistin played a role in increasing membrane permeability under the combination treatment. Nevertheless, a small number of DEGs associated with cell motility, electron transport chain, oxidative phosphorylation, C4-dicarboxylate transporter, and drug/metabolite exporter showed a significant downregulation, which suggests that it might only be a weak influence involving the restoral of azithromycin susceptibility and needs to be studied further.

**Fig 4:**
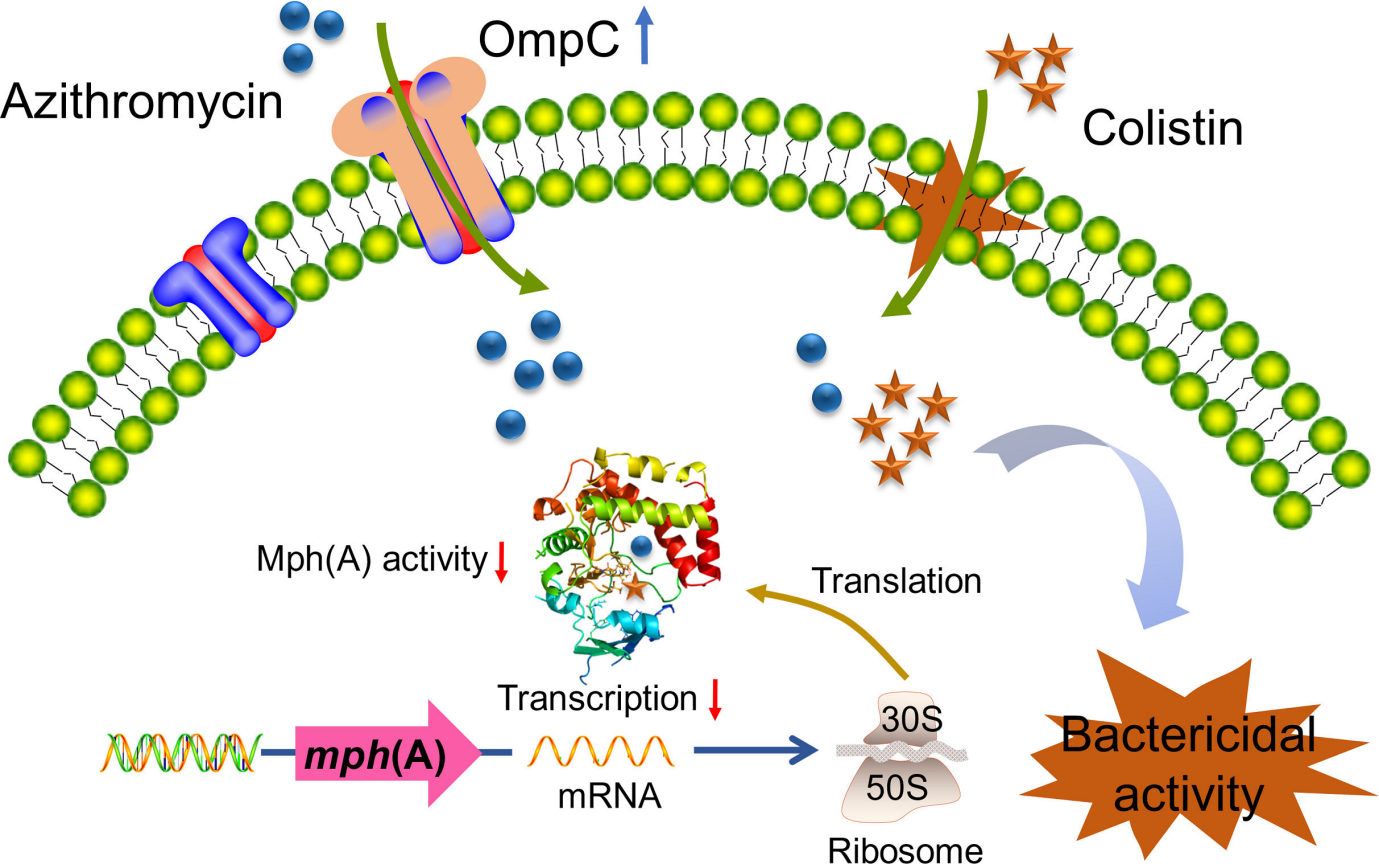
Schematic diagram of the restoral of azithromycin susceptibility including upregulation of the *ompC* gene and the suppression of the mph(A) activity after *E. coli* T28R treated with azithromycin plus colistin. The upregulation of the *ompC* gene and azithromycin accumulation caused by colistin provided the opportunity for remodeling the conformation of the mph(A) by colistin along with azithromycin, and the *mph*(A) gene was significantly downregulated, leading to the suppression of the Mph(A) activity and finally showing bactericidal activity. The blue up arrow expresses the upregulation of *ompC* gene, and the red down arrows express the downregulation of the *mph*(A) gene and the suppression of the Mph(A) activity.

The phosphotransferase Mph(A), encoded by *mph*(A) gene, was found to efficiently inactivate both 14-membered lactone macrolides (erythromycin) and the 15-membered lactone macrolide (azithromycin) (15). Notably, the addition of colistin resulted in the downregulation of the *mph*(A) gene and further led to the conformational change of the Mph(A) protein, suppressing the phosphotransferase activity and resulting in the failure modification of the macrolide structure (9). For example, the residue ASP-200 shows the function to orient the target hydroxyl group of the macrolide substrate (16), but colistin can also compete with the ASP-200 position forming a tight complex with the Mph(A) protein in the presence of azithromycin. Therefore, it is conceivable that colistin firstly had an important effect on the improvement of membrane permeability causing intracellular drug accumulation and then the suppressive activity of the *mph*(A) function along with azithromycin, finally exhibiting bactericidal efficacy (Fig. 4).

In conclusion, our study provided a novel insight into the reverse of azithromycin resistance. Our data suggested that the synergistic activity of azithromycin with colistin is not exclusively related to the downregulation of *mph*(A) gene and conformational remodeling; it is also related to the increased accumulation of azithromycin partly because of the upregulation of membrane porin gene *ompC*. Investigations on the regulation of bacterial porins would be significant in clinical isolates under the selective pressure of antibiotics.

## MATERIALS AND METHODS

### Strains, culture conditions, and antimicrobial susceptibility testing

*E. coli* T28R was preserved in our lab at Henan Agricultural University. BL21 (DE3) was purchased from Beijing Tsingke Biotech Co., Ltd. (no. TSC-E01, China). These strains were cultured in Luria-Bertani (LB) broth or Maconkey agar plates (Beijing Land Bridge Technology), unless they have special descriptions. Antibiotics were obtained from Henan Muxiang Veterinary Pharmaceutical Co., Ltd. (China). Antimicrobial susceptibility testing was performed according to Clinical and Laboratory Standards Institute guidelines (17).

### Transcriptome analysis

Transcriptome analysis was performed according to a previously described process (18). *E. coli* T28R was cultured overnight and diluted 1:100 in fresh LB broth to obtain an OD_600_ of 0.4. The cultures were treated with 2-µg/mL azithromycin or in combination with 1-µg/mL colistin for 1 h, and then the bacteria were washed with phosphate-buffered saline (PBS) three times and precipitated at 8,000 rpm for 10 min at 4°C. The precipitates were quickly frozen in liquid nitrogen and subjected to transcriptome sequencing by Majorbio Bio-pharm Technology Co., Ltd., Shanghai, China, and the sequencing data were analyzed using the Majorbio Cloud Platform (www.Majorbio.com).

### Reverse transcription-quantitative PCR

Some membrane-related DEGs (such as *tolA*, *tolR*, *eptB*, *lpxP*, and *ompC*) were verified by RT-qPCR using the PrimeScript RT Reagent Kit with gDNA Eraser (TaKara, Beijing, China) and were analyzed using a 7500 Real-Time PCR system (Applied Biosystems, Thermo Fisher Scientific, Inc.). The primer sequences are listed in Table S1. The relative expression of these selected genes was compared to the expression of the *16S rRNA* gene. Relative mRNA levels were analyzed using the 2^-ΔΔCT^ method and *t*-test (19). All the tests were performed in triplicate.

### Azithromycin accumulation analysis

The accumulation of azithromycin in *E. coli* T28R was measured using an Azithromycin ELISA Test Kit (Lvshiyuan, Shenzhen, China) according to the manufacturer’s instructions. In brief, the isolate T28R was grown overnight in Mueller-Hinton Broth (MHB, Beijing Land Bridge Technology) and then washed and suspended in PBS to obtain an OD_600_ of 0.5. The suspension was incubated with 8-µg/mL azithromycin and gradient concentrations of colistin for 1 h. After the bacteria were harvested and washed three times with PBS, the bacterial precipitates were lysed by lysozyme and subjected to determining the intracellular azithromycin levels.

### Overexpression analysis of *ompC* gene

The *ompC* gene was amplified by PCR from genomic DNA of *E. coli* T28R using Q5 High-Fidelity DNA Polymerase (New England Biolabs, Ipswich, MA, USA) and was cloned into plasmid pET21b using HindIII and XhoI (TakaRa, Beijing, China) restriction sites. The used primer set containing HindIII and XhoI restriction sites was listed in Table S1. The target PCR products and expression vector pET21b were respectively digested with the restriction enzymes, and then these digested products were ligated with each other using T4 DNA ligase (TakaRa, Beijing, China), generating pET21b-*ompC*. The recombinant plasmid pET21b-*ompC* was transformed into *E. coli* BL21 (DE3) chemically competent cells. The transformants were then cultured overnight in LB broth supplemented with 100-µg/mL ampicillin at 37°C, diluted 1:100 in fresh LB medium to grow to obtain approximately 1.0 × 10^6^ CFU/mL, subjected to susceptibility testing of colistin and azithromycin.

### Molecular docking

Molecular docking was employed to investigate the action model between colistin and macrolide 2′-phosphotransferase Mph(A) by using AutoDock 4.2.6 software. The three-dimensional (3D) structural colistin was obtained from the PubChem database (https://pubchem.ncbi.nlm.nih.gov/, Compound CID: 5311054). The Mph(A) proteins were downloaded from RCSB Protein Data Bank (PDB) (https://www.rcsb.org/), including Mph(A) (PDB ID: 5IGH) and Mph(A) containing azithromycin ligand (PDB ID: 5IGI). After binding pockets were identified using the ProteinsPlus server (Universität Hamburg), the grid boxes of the protein receptors (5IGH and 5IGI) were respectively adjusted to 34, 68, and 86 Å in the x, y, and z orientations. Other parameters were set as default. AutoDock program was run with Lamarckian Genetic Algorithm, and the interacting amino acid residues were visualized using AutoDock Tools and PyMOL 2.5 (https://pymol.org/2/).

## Data Availability

The complete nucleotide sequence of the chromosome in *E. coli* T28R has been deposited in GenBank under accession number CP049353. All the raw transcriptome data were deposited into the NCBI database as a BioProject (accession no. PRJNA968067).

## SUPPLEMENTAL MATERIAL

Supplemental material spectrum.03918-23-s0001.docx Fig. S1 and S2; Table S1.

ASM does not own the copyrights to Supplemental Material that may be linked to, or accessed through, an article. The authors have granted ASM a non-exclusive, world-wide license to publish the Supplemental Material files. Please contact the corresponding author directly for reuse.
