# Supplementary material for: Upregulation of outer membrane porin gene ompC contributed to enhancement of azithromycin susceptibility in multidrug-resistant Escherichia coli
Source: Microbiol Spectr. 2024 Mar 5;12(4):e03918-23. doi: 10.1128/spectrum.03918-23 (PMC10986464; doi:10.1128/spectrum.03918-23)
Supplement: Supplemental material — Fig. S1 and S2; Table S1. [file spectrum.03918-23-s0001.docx]

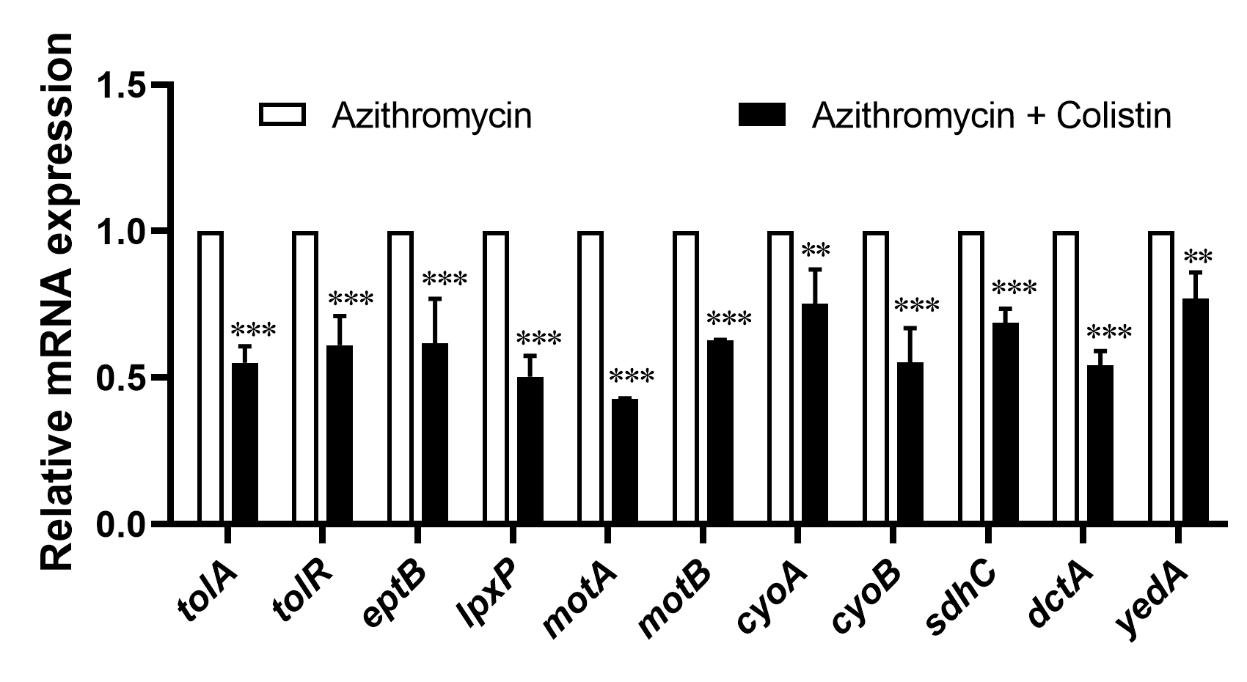


Fig. S1 RT-qPCR analysis. Relative expression of representative genes of *E. coli* T28R after treatment with azithromycin alone or in combination of colistin. Data are presented as mean ± SD and unpaired t-test was used to calculate *P*-values (**P* < 0.05, ***P* < 0.01, ****P* < 0.001).


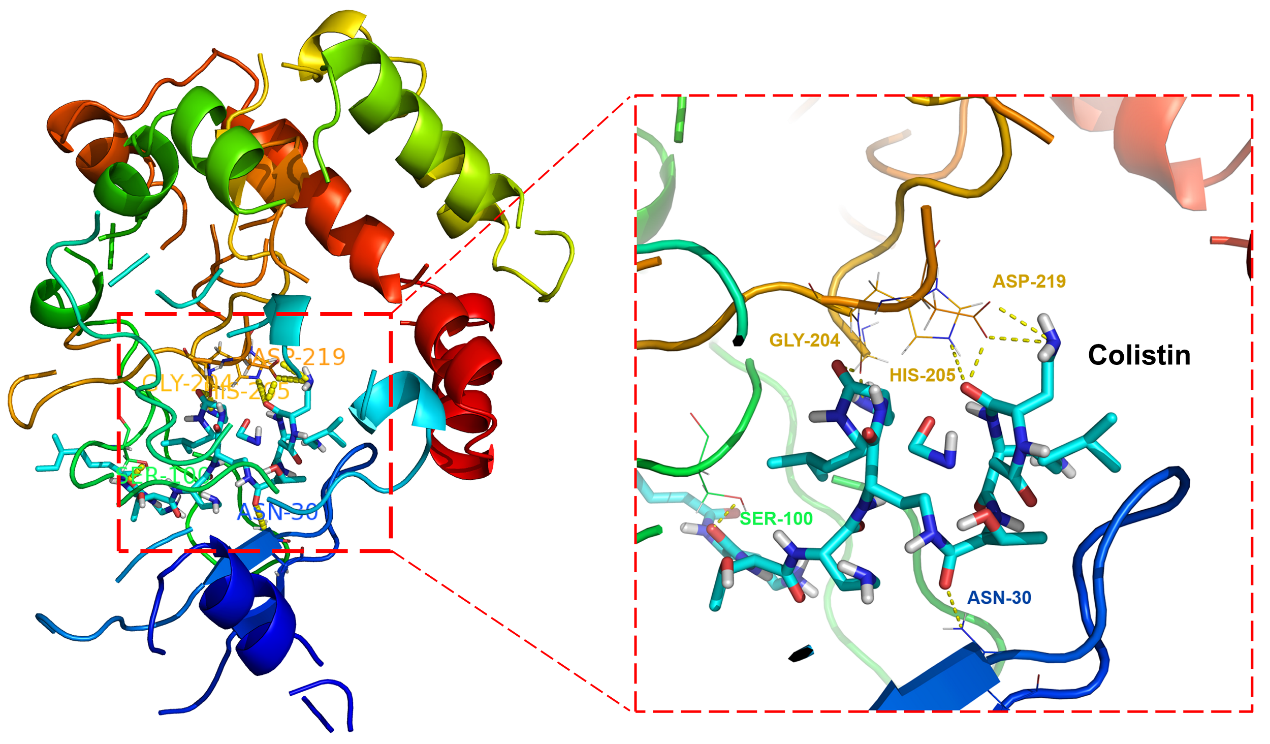


Fig. S2 Binding model of colistin and the Mph(A) protein (5IGH). Red dashed rectangles magnified showed the specific interacting modes between colistin and amino acid residues in the docking pocket. Yellow dashed represents hydrogen bonds.

Table S1 Primer set used in this study

| Genes | Sequencing (5’~3’) | Product (bp) | Use |
| --- | --- | --- | --- |
| *tolA* | F: ctcagcttctgctttggctt  R: tgcggcactgaagaagaaag | 103 | RT-qPCR |
| *tolR* | F: aggcgatctttctcaaccac  R: tctgccagacgctactgaat | 111 |  |
| *eptB* | F: gtggtcattggttatggcat  R: tgcactaacggcgattaacc | 108 |  |
| *lpxP* | F: gagctgcctttacgaccata  R: ctaacaaagcgatgatcggc | 112 |  |
| *motA* | F: ggatgaagagattgagacgc  R: tgaacctaacgcgtgaacga | 130 |  |
| *motB* | F: gtgttacgtgtcgtcggcat  R: catgcaaaatggcctgttcg | 124 |  |
| *cyoA* | F: ttccttgcagtactgacctg  R: ttccgggtagatgaagaacc | 129 |  |
| *cyoB* | F: cgacgcttaccggtatcaac  R: gtcagaattgggaaggaagc | 139 |  |
| *sdhC* | F: cgtttacccgcttcgaatgt  R: agcaagcttccgcgattatg | 148 |  |
| *dctA* | F: taccatcggtaaatacggcg  R: gaagatactgaaaccagtcg | 133 |  |
| *yedA* | F: tggcaatacccacccattcc  R: cggttgccgaacatcaaaatg | 132 |  |
| *ompC* | F: caacttcatgcagcagcgtg  R: ttgccctggtactgaacagc | 102 |  |
| *opgE* | F: agtcgaccgaaatcctgacg  R: tgaataccagacacaggctg | 147 |  |
| *mph*(A) | F: tcgaagactcgactgcgatg  R: gaaatggggacggcatgca | 130 |  |
| *ompC* | F: (5’ -HindⅢ) CCAAGCTTcgccattccgcaataatctt  R: (3’ -XhoI) CCctcgagTCAtacgtgattatcctcatgcg | 1446 | Clone of *ompC* gene |

Note: Introduced restriction enzyme sites are underlined.
